# The Molecular Mechanism of Action of Artemisinin—The Debate Continues

**DOI:** 10.3390/molecules15031705

**Published:** 2010-03-12

**Authors:** Paul M. O’Neill, Victoria E. Barton, Stephen A. Ward

**Affiliations:** 1 Department of Chemistry, University of Liverpool, Oxford Street, Liverpool L697ZD, UK; E-Mail: V.Barton@liverpool.ac.uk (V.E.B.); 2 Liverpool School of Tropical Medicine, Pembroke Place Liverpool L35QA, UK; E-Mail: saward@liverpool.ac.uk (S.A.W.)

**Keywords:** artemisinin, bioactivation, molecular targets, antimalarial, antitumor agent

## Abstract

Despite international efforts to ‘roll back malaria’ the 2008 World Malaria Report revealed the disease still affects approximately 3 billion people in 109 countries; 45 within the WHO African region. The latest report however does provide some ‘cautious optimism’; more than one third of malarious countries have documented greater than 50% reductions in malaria cases in 2008 compared to 2000. The goal of the Member States at the World Health Assembly and ‘Roll Back Malaria’ (RBM) partnership is to reduce the numbers of malaria cases and deaths recorded in 2000 by 50% or more by the end of 2010. Although malaria is preventable it is most prevalent in poorer countries where prevention is difficult and prophylaxis is generally not an option. The burden of disease has increased by the emergence of multi drug resistant (MDR) parasites which threatens the use of established and cost effective antimalarial agents. After a major change in treatment policies, artemisinins are now the frontline treatment to aid rapid clearance of parasitaemia and quick resolution of symptoms. Since artemisinin and its derivatives are eliminated rapidly, artemisinin combination therapies (ACT’s) are now recommended to delay resistance mechanisms. In spite of these precautionary measures reduced susceptibility of parasites to the artemisinin-based component of ACT’s has developed at the Thai-Cambodian border, a historical ‘hot spot’ for MDR parasite evolution and emergence. This development raises serious concerns for the future of the artemsinins and this is not helped by controversy related to the mode of action. Although a number of potential targets have been proposed the actual mechanism of action remains ambiguous. Interestingly, artemisinins have also shown potent and broad anticancer properties in cell lines and animal models and are becoming established as anti-schistosomal agents. In this review we will discuss the recent evidence explaining bioactivation and potential molecular targets in the chemotherapy of malaria and cancer.

## 1. Introduction 

Global warming has impacted upon malaria disease burden making it difficult to predict future disease patterns [1]. However the most critical problem facing the treatment of malaria is the development of resistance to classical quinoline antimalarial compounds such as chloroquine and antifols [2]. A significant discovery program by Chinese chemists in the 1970’s; ‘project 523’ has provided one of the most potent and effective antimalarials to date, artemisinin (**1**) [3]. Artemisinins are effective not only against multi-resistant strains of *P. falciparum*, but have broad stage specificity against the *Plasmodium* life cycle including activity throughout the asexual blood stages[4] and also the sexual gametocyte stages which may reduce the spread of the disease in areas of low transmission [5]. 

**Figure 1:**
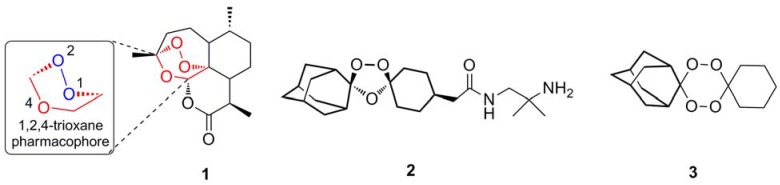
Antimalarial endoperoxides.

Representing a new class of antimalarial agents, artemisinin is a sesquiterpene trioxane lactone whose endoperoxide bridge is essential for antimalarial activity. Although the precise mechanism of action is still highly controversial [6]; the endoperoxide pharmacophore alone has stimulated the development of several different classes of totally synthetic endoperoxides including the trioxolane OZ277 (**2**) [7] and the tetraoxane **3** [8] (Figure 1). Understanding the mechanism of action of this class of drugs will allow the prediction of potential resistance mechanisms and aid targeted design of future antimalarial agents.

Artemisinins have also been investigated for their antiproliferative effects against a wide range of cancer cell lines [9]. The promising *in vitro* profiles of several semi-synthetic analogues has prompted the call for more appropriate clinical studies to be performed [10,11]. In this review we will consider the different views regarding how artemisinin is first activated and its proposed molecular targets. The effect of artemisinin in cancer cells will also be considered with respect to similarities and differences to their action in malaria chemotherapy, factors which may be important in elucidating potential common mechanisms of action.

## 2. Activation of Artemisinin 

### 2.1. Bioactivation in parasites

**Figure 2:**
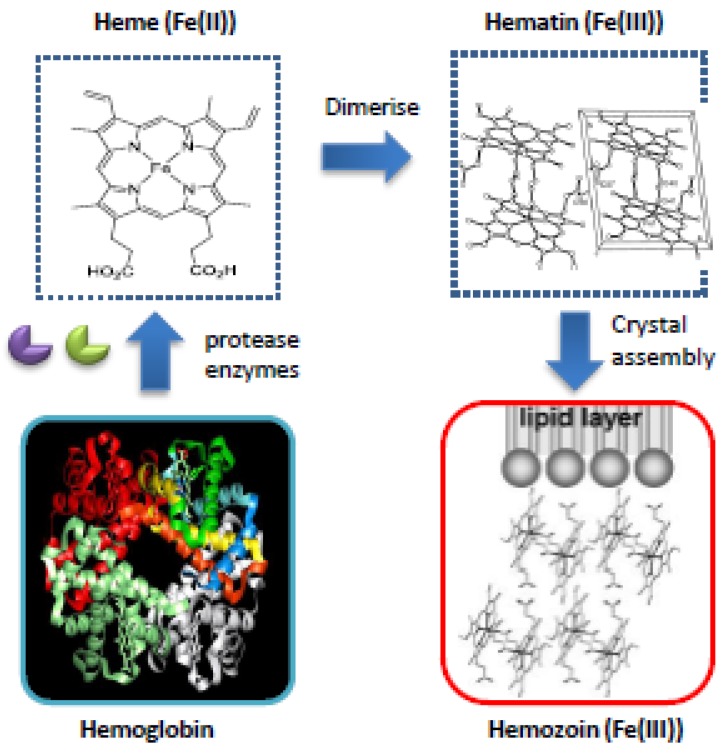
Detoxification of hemoglobin: toxic hematin (formed by hydrogen bonding of heme monomers) [12] is converted by the parasite to an insoluble non-toxic compound called hemozoin (it has recently been suggested that the propionate group of each Fe(III)PPIX molecule coordinates to the Fe(III) centre of its partner) [13].

Within the malaria parasite host hemoglobin is degraded by a series of protease enzymes to release peptides and amino acids required for development and to create space within its digestive vacuole. During this process a build up of hematin occurs which is potentially toxic to the parasite (Figure 2). To circumvent this toxicity, the parasite has developed a mechanism whereby hematin undergoes biomineralization to form insoluble non-toxic hemozoin (malaria pigment).

One of the first studies completed by the Meshnick group [14] suggested that the bioactivation of 1,2,4 trioxanes is triggered by iron (II) to generate toxic activated oxygen. The selectivity of artemisinin towards parasite-infected erythrocytes over normal erythrocytes was rationalised by the iron dependent bioactivation of the endoperoxide bridge. Since these initial findings two models of ring opening have been suggested that differ by their dependency on iron and involvement of carbon centred radicals.

#### 2.1.1. Reductive scission model

**Figure 3:**
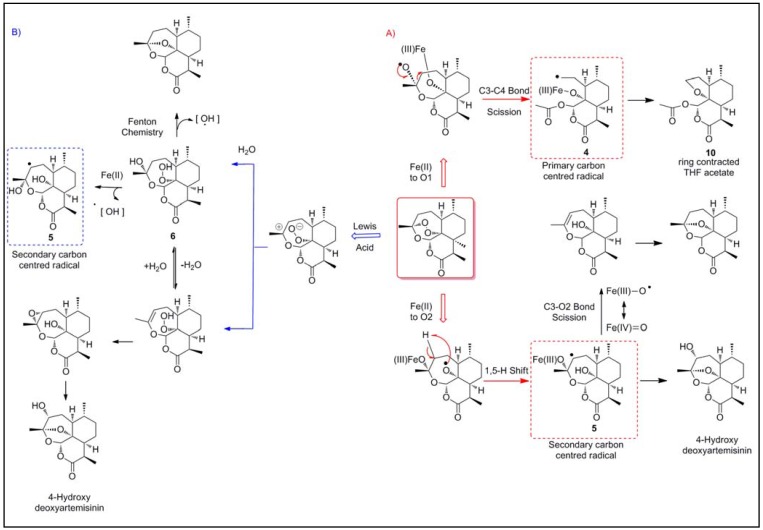
Bioactivation of artemisinin; A) Reductive scission model; B) Open peroxide model.

Early work by Posner [15,16,17] and Jefford [18,19] proposed that these oxygen centred radicals subsequently rearrange to form carbon centred radicals, although the nature of the proposed radical and the mechanistic pathways giving rise to their formation were different in each case. Low valent transition ions (ferrous heme or non heme exogenous Fe^2+^) were found to bind to artemisinin and after subsequent electron transfer induce reductive scission of the peroxide bridge to produce oxygen centred radicals which rearrange to give carbon centred radicals (Figure 3 A). 

Due to the unsymmetrical nature of the endoperoxide bridge, iron was found to interact with the peroxide in different ways to produce either a primary carbon centred radical or a secondary carbon centred radical. Both primary and secondary radicals, **4** and **5** [20,21] have been efficiently spin-trapped by electroparamagnetic resonance spin trapping techniques after being activated by iron [22]. 

#### 2.1.2. Open peroxide model

The alternative model suggests that ring opening is driven by protonation of the peroxide or by complexation by Fe^2+^ (Figure 3 B). Haynes and co-workers have proposed that iron acts as a Lewis acid to facilitate ionic, rather than radical bioactivation of the artemisinins (which, it is proposed, are too short-lived to have any intermolecular interaction) [23]. In addition, it has also been suggested that non-peroxidic oxygen plays a role in facilitating ring opening of the peroxide to generate the open hydroperoxide [24,25,26]. The oxygen atom provides stabilization of the positive charge and, according to transition state theory, lowers the energy required for ring opening. Heterolytic cleavage of the endoperoxide bridge and subsequent capture of water leads to the formation of an unsaturated hydroperoxide **6**, capable of irreversibly modifying protein residues by direct oxidation. Subsequent Fenton degradation of the hydroperoxide **6** produces a hydroxyl radical, a species that can subsequently oxidize target amino acid residues. To support this theory artemisinin has been shown to mediate *N*-oxidation of tertiary alkylamine derivatives *via* the intermediacy of such a ring opened peroxide form of artemisinin [25]. This alternative mechanism may have the potential to produce a whole host of reactive oxygen species that may have implications for the antimalarial activity of these compounds.

#### 2.1.3. Iron-Dependent bioactivation *vs.* heme-dependent bioactivation in parasites

**Figure 4:**
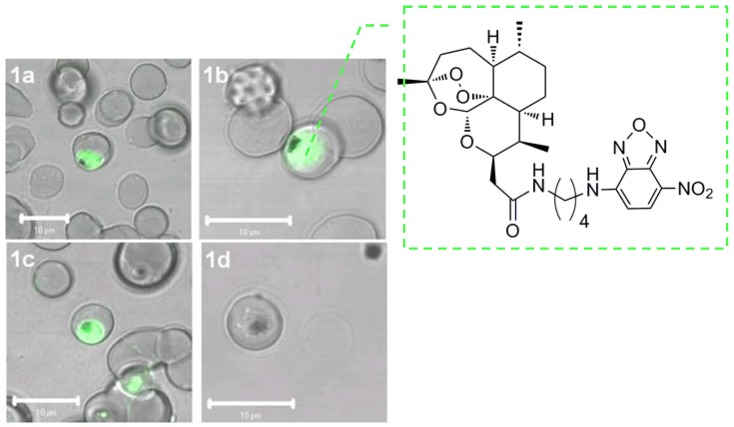
Confocal microscope images of parasite-infected red blood cells incubated with fluorescent artemisinin nitrobenzodiazole (NBD) conjugate without iron chelator DFO before (1a) and after wash (1b), and with DFO (100 µM) before (1c) and after wash (1d) [27].

The species originally thought responsible for bioactivation was heme iron, as this form of iron is in abundance within the parasite due to the degradation of hemoglobin releasing soluble heme. However, intracellular iron (II) and iron (III) occur also in equilibrium inside the food vacuole of the parasite, where digestion of hemoglobin takes place. Using semi-synthetic artemisinins in combination with iron chelators (selective for non-heme sources of iron) antagonizes the efficacy of artemisinin. Figure 4 shows a marked accumulation of fluorescently labelled drug within the parasite cytoplasm and in the food vacuole, either in the presence (1c–1d) or absence of the iron chelator desferrioxamine (DFO) (1a–1b). In the presence of DFO the drug is completely washed out (1d), suggesting that iron is required for the drug to become covalently bound to parasite macromolecules. These observations suggest that the labelled compounds are first being accumulated by the parasite and then activated by a non-heme chelatable iron source [27]. 

A similar iron dependency was found for several classes of endoperoxide antimalarials including totally synthetic trioxolanes and tetraoxanes [27]. However the fact that the endoperoxide activity was antagonized but not abolished [28], has prompted some researchers to suggest a competitive pathway [23,29]. In addition, while it is clear that DFO is an effective inhibitor of iron-related oxidative stress by chelation, it is also the case that DFO can impact on oxidative reactions independently of its ability to chelate iron [30]. A recent study suggests that when used *in vivo*, DFO can also act *via* iron-independent pathways by at least two mechanisms indirectly associated with iron chelation to protect against oxidative damage [31,32]. 

A recent study analysing the reactions of artemisinin with different redox forms of heme, ferrous iron, and deoxygenated and oxygenated hemoglobin under similar *in vitro* conditions found that heme reacted with artemisinin much more efficiently than the other iron-containing molecules, supporting the role of redox active heme as the primary activator of artemisinin [33]. The stability of peroxide antimalarials with intact oxyhemoglobin (most abundant form of iron in humans), and reactivity with free heme, may explain the selective toxicity of these antimalarials toward infected, but not healthy, erythrocytes [34]. This heme-dependent theory conflicts with reports that artemisinins are also active against early ring stage parasites containing very little hematin [35]. In addition, artemisinin is active against other parasite species such as *Toxoplasma* and *Babesia* that do not contain hematin [36,37]. 

However it was recently shown using fluorescence microscopy and image analysis that release of hemoglobin from heme is initiated in ring stage parasites and would be available as a source of iron based activator which is consistent with the activity of artemisinin at this stage [38]. 

### 2.2. Bioactivation in tumor cells

Woerdenbag *et al* were the first to document the cytotoxicity of artemisinins to tumour cells [39]. It was found that artemisinin had activity in the micromolar range, whereas semi-synthetic analogues such as sodium artesunate had more potent activities in the low micromolar range. Further studies suggested that these compounds exert their effect on tumour cells by growth inhibition [40,41]. This cytotoxicity was also shown to be endoperoxide-dependent [42]. Selective activation of artemisinin by tumor cells has led to proposal of an iron dependent hypothesis due to the understanding that tumor cells maintain a high intracellular iron concentration to sustain continued proliferation in addition to an increased capacity to synthesize heme [43]. 

**Figure 5:**
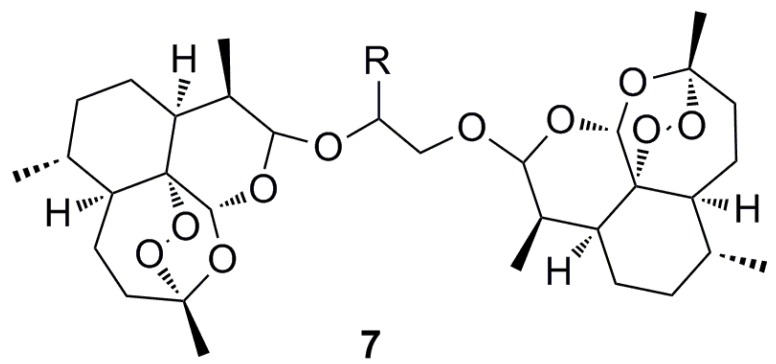
artemisinin dimer **7**.

Cancer cells exposed to artemisinin demonstrate decreased proliferation, increased levels of oxidative stress, induction of apoptosis and inhibition of angiogenesis [9]. Interestingly, artemisinins have also shown cytotoxicity against drug and radiation resistant cell lines suggesting a different mechanism to traditional anti-cancer therapies [44]. The activation of antitumor immune responses is believed to suppress tumor growth therefore suppression of these responses by artemisinins may counteract anticancer activity. However recent results using a transgenic mouse melanoma model indicate that the cytostatic and apoptotic effects of artesunate are not diminished by simultaneous immunosuppression [45]. Whereas the monomeric forms of artemisinin have superior activity in the treatment of malaria, it is the dimeric forms of artemisinin, such as **7** (Figure 5), that have shown enhanced anticancer activity [46]. 

With respect to bioactivation of artemisinin in tumor cells the actual mechanism is still unclear however the current consensus involves the iron(II)-mediated release[47] of reactive oxygen species (ROS) [48] and/or carbon centered radicals [49]. Both may play an important role in inducing DNA damage, mitochondrial depolarisation and apoptosis. However other factors may also come into play such as the ability of the tumor cell to transport ferrous iron and maintain supplies. In a study to explore the response of iron transporter proteins of tumor cells to artesunate, nearly a third of tumor cell lines showed no enhancement or even decreased activity upon addition of ferrous iron [50]. Enhancement of artesunate response by ferrous iron was found to depend on the expression of two genes: the iron-binding transferrin receptor (TfR) and ATP-binding transporter (ABCB6). Hence, pre-therapeutic detection of TfR and ABCB6 expression may predict the response of tumor cells towards artesunate. Consequently this result could be exploited for individualized tumor therapy with artemisinins.

Recently several studies have identified heme as the mediator of cytotoxicity of artemisinin both in its monomeric and dimeric form. Stimulation of the synthesis of heme within cancer cells using promoters was shown to increase cytotoxicity of dihydroartemisinin (DHA) whereas inhibition of heme synthesis using inhibitors caused a decrease in cytotoxicity [51]. To rule out the possibility that intracellular ‘free’ iron is involved, holotransferrin (diferric transferrin) was added as an additional source of intracellular iron and as expected the cytotoxic effect of DHA dramatically increased. Inhibition of heme synthesis by succinyl acetone negated the effect of the holotransferrin, suggesting the newly acquired iron indirectly increases cytotoxicity by increasing heme biosynthesis. 

Further evidence for the involvement of heme in the anticancer activity was shown using artemisinin dimers in combination with cobalt and tin protoporhyrin, modulators of heme oxygenase (HMOX1, a heme degradation catalyst) [46]. Cobalt protoporhyrin, a HMOX1 inducer abolished the activity of an artemisinin dimer whereas tin protoporhyrin, a HMOX1 inhibitor enhanced the activity of the dimer. Pre-treatment of cells with iron chelators DFO or exogenous hemin decreased the activity of the artemisinin dimer. 

## 3. Potential targets of the artemisinins

### 3.1. Proposed parasite molecular targets 

#### 3.1.1. Heme

Alkylation of heme by artemisinin was first reported by Meshnick [14,52] who identified heme-drug adducts by mass spectrometry. The *in vitro* reaction of artemisinin and heme, in the presence of red cell membranes was also shown to cause oxidation of protein thiols [53]. 

**Figure 6:**
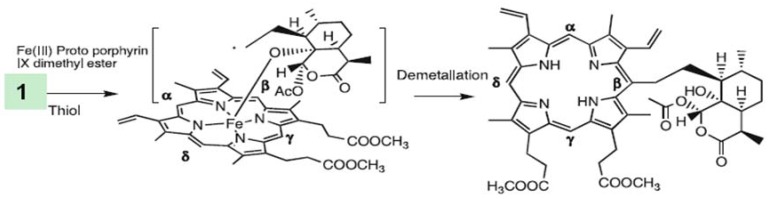
Alkylation of a heme model (the dimethyl ester of heme) by a primary carbon centered radical derived from bioactivation of artemisinin (**1**). Adducts were also obtained from α and δ carbon atoms [54].

Artemisinin was later shown to be capable of alkylating a heme model at the α, ß and δ carbon atoms (Figure 6) [54]. Studies with totally synthetic trioxolanes [55] and tetraoxanes also support this mechanism, indicating alkylation of heme *in vitro* as identified by LC-MS.

Robert *et al.* [56] also found heme-artemisinin adducts in the spleen and urine of mice infected with *Plasmodium vinckei* and treated with artemisinin. In the urine, the hydroxylated and glucuronyl-conjugated derivatives of covalent adducts were found and identified by LC-MS. Recently the same group have shown that trioxaquines (drug hybrids containing both an aminoquinoline moiety, as in chloroquine and a synthetic 1,2,4-trioxane entity as an artemisinin mimic) afforded covalent heme-drug adducts that were detected in the spleens of *Plasmodium* infected mice [57]. 

While these results suggest that interference of hematin formation and the accumulation of heme is a possible mechanism it has also been contested since the *in vivo* evidence is somewhat open to interpretation. In studies with infected mice it has been claimed that detection of heme-drug adducts (as glucuronyl-conjugated derivatives) is evidence that this alkylation process is key in the *in vivo* mechanism of action. However, in infected mice the progress of infection ultimately leads to hematin deposition in both the liver and the spleen. Thus it cannot be ruled out that the heme-drug adducts originate from an ex-vivo parasite interaction within the organs.

#### 3.1.2. Protein alkylation

Since artemisinin is sensitive to steric effects it was suggested that its target may be a particular protein or enzyme. A number of studies have shown radiolabeled artemisinin reacting covalently with several parasitic proteins [52,58]. Autoradiograms of SDS-polyacrylamide gels showed that six malarial proteins are radiolabelled by three different endoperoxides; arteether, dihydroartemisinin (DHA) and arteflene. The labelling occurred at physiological concentration of the drug and was not stage or strain specific. In addition, the labelled proteins were not the most abundant proteins seen on coomassie stained gels. The uninfected erythrocytes and controls treated with the inactive analogue deoxyarteether failed to label any proteins [59]. Another study demonstrated specifically the *in situ* and *in vitro* covalent reaction between artemisinin and a 25 KDa translationally controlled tumour protein (TCTP) homolog [60]. *In vitro*, this alkylation appears to be dependent on heme and the homolog is able to bind heme with modest affinity. It was suggested that the cysteine of this protein is necessary for this mechanism by serving as a source of electron for the heme-mediated activation of the drug.

In a different study, artemisinin also alkylated various proteins *in vitro* [52]. Between 5–18% of added drug bound to hemoproteins such as catalase, cytochrome c and hemoglobin, however the drug did not react with heme free globin. In addition, the *in vitro* alkylation of human albumin by artemisinin is well documented and is shown to react on both the thiol and amino moieties *via* iron dependent and independent reactions [58]. Further work in this area has identified cysteine protease adducts of artemisinin derived radicals suggesting that general alkylation of cysteine residues may be involved in the mechanism of action by interfering with protein function [61]. Artemisinins have also been shown to inhibit the falcipains, a papain family cysteine protease that aid hemoglobin degradation. This mechanism of protease inhibition was shown to increase in the presence of heme [62]. 

#### 3.1.3. Inhibition of PfATP6

**Figure 7:**
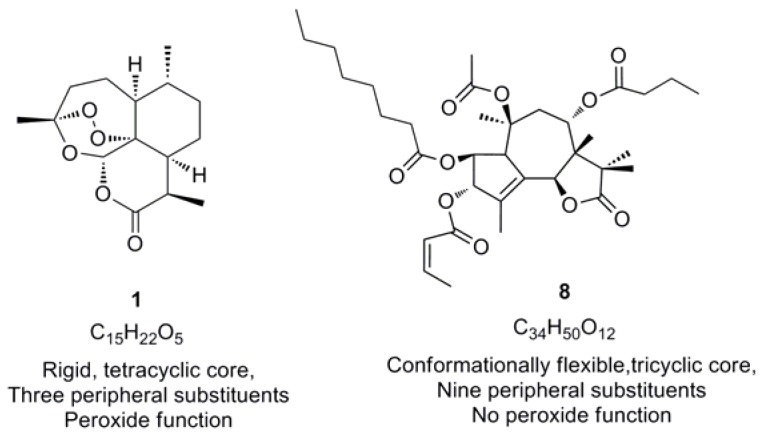
Comparison of the structural and chemical features of sequiterpene lactones artemisinin (**1**) and thapsigargin (**8**) [63].

Thapsigargin (**8**) is a sesquiterpene lactone and a highly selective inhibitor of a mammalian Ca^2+^ transporting ATP-ases (SERCA – sarco/endoplasmic reticulum membrane calcium ATP-ase.). SERCA’s role is to reduce cytosolic free calcium concentrations by actively concentrating Ca­­­­­^2+^ into membrane bound stores, an activity critical to cellular survival. It was therefore reasoned that, because thapsigargin and artemisinin are both sesquiterpene lactones, they would behave in a similar manner towards SERCA-type enzymes [28]. Surprisingly they did behave similarly, despite marked differences in their chemical and molecular structure. Interestingly, thapsigargin lacks the important pharmacophore responsible for the antimalarial activity of artemisinin (Figure 7).

*P. falciparum* has only one enzyme orthologous to SERCA; *Pf*ATP6ase. To test the SERCA hypothesis *Pf*ATP6 was expressed in frog’s eggs (*Xenopus laevis* oocytes). It was found that both artemisinin and thapsigargin inhibit the enzyme irreversibly whereas deoxyartemisinin, quinine and chloroquine had no effect. This mechanism was also highly specific; no other malarial transporters were affected (including the non-SERCA Ca^2+^ ATPase *Pf*ATP4) [28]. 

To confirm that the antiparasitic activity and inhibition of the enzyme by artemisinin was iron dependent, parasite–infected red blood cells were incubated with desferrioxamine (DFO, an iron chelator) in combination with artemisinin and/or thapsigargin. DFO abolishes the inhibitory activity of artemisinin on *Pf*ATP6 but did not alter the inhibitory properties of thapsigargin suggesting that artemisinins act by inhibiting *Pf*ATP6 after activation by iron [28]. Isobologram analysis was also used to confirm that thapsigargin and artemisinin interact with the same target. When both used simultaneously, thapsigargin antagonises the activity of artemisinin suggesting competition for *PfATP6* [28]. Interestingly, the target: *PfATP6* resides in the endoplasmic reticulum of the parasite, not the food vacuole. A fluorescent artemisinin was synthesized which irreversibly labelled the structures of the cytoplasm rather than inside the food vacuole. However, it was later shown that tagging artemisinin using a different fluorescent conjugate nitrobenzodiazole, a fluorophore not quenched by heme, labels both the food vacuole and the cytoplasm [27]. 

Further evidence supporting this theory is the effect of artemisinin on the *Toxoplasma gondii* SERCA homolog. Results demonstrate that artemisinin perturbs calcium homeostasis in *T. gondii*, supporting the idea that calcium dependent ATPases are potential drug targets in parasites. Soon after the discovery of this potential target it was suggested that artemisinin resistance could be introduced by altering a single amino acid in *plasmodial* SERCA [64]. This amino acid (L263E) is thought to act as a ‘gatekeeper’ influencing access or binding of artemisinins to this site modulate which in turn dictates the sensitivity to artemisinin *in vitro* [65]. However a genetic analysis of artemisinin resistant strains of *P. falciparum* and *P. chabaudi* (rodent malaria) found no such mutation in the ATP6 gene contradicting this hypothesis [66]. 

Since this work was published there have been several studies which contradict the previously reported SERCA inhibition. Since SERCA is an endoplasmic reticulum (ER) protein, artemisinin was expected to have a marked effect on the morphology of the ER. However it was found that thapsigargin had a major effect on ER morphology whereas at ten times its IC_50_, artemisinin had no significant effect on the ER. Contrary to previous reports, studies repeated by Crespo *et al.* also indicated that there was no antagonism between thapsigargin and artemisinin [67]. 

A synthetic trioxolane **2** (OZ277) was also shown to be a very weak inhibitor of *Pf*ATP6. Recent studies to determine the possible mechanisms of action of OZ277 focused on its ability to inhibit the proposed target *PfATP6.* OZ277 is two orders of magnitude less potent (apparent half-maximal inhibitory constant, *K_i_* = 7,700 nM) than artemisinin (*K_i_* = 79 nM) against *Pf*ATP6 [68]. This is an interesting development especially since OZ277 antagonizes the activity of artemisinin. Recently the 3D structure of *Pf*ATP6 was also modelled and used to predict binding affinities of artemisinin (in addition to other antimalarials such as trioxolanes, tetraoxanes, trioxaquines and quinolones) however no correlation was found between affinity of the compounds for *Pf*ATP6 and *in vitro* antimalarial activity [69]. 

#### 3.1.4. Parasite membranes 

Recently artemisinin was shown to accumulate within neutral lipids and cause parasite membrane damage. This effect was endoperoxide dependent since analogues lacking the endoperoxide moiety failed to label neutral lipid bodies or induce oxidative membrane damage [70]. This work is consistent with a previous study where disruption of the digestive food vacuole membrane by artemisinin was an important initial site of endoperoxide antimalarial activity whereas novel synthetic endoperoxides cause the early accumulation of endocytic vesicles [67]. In another study however tetraoxanes were shown to cause oxidative degradation of phospholipids whereas the same effect was not evident using artemisinin [71]. 

#### 3.1.5. Mitochondria

Components of the electron transport chain in yeast have shown to be susceptible to artemisinin [72]. However recent analysis of mitocondrial function in endoperoxide treated parasitized erythrocytes showed no obvious effect on the morphology of the mitochrondion, suggesting that loss of mitrocondrial function is not an early event in the action of artemisinin or synthetic endoperoxides [67]. 

### 3.2. Potential molecular targets in tumor cells

There is an inverse correlation between activity of artesunate and mRNA expression for antioxidant genes such as catalase, superoxide dismutase II, thioredoxin reductase, γ-glutamylcysteine synthase and several members of the glutathione-S-transferase (GST) family [48]. Over-expression of enzymes associated with oxidative stress have shown to reduce susceptibility of tumor cells to artemisinins [44,73,74]. 

Although numerous studies have shown mechanistic pathways that can selectively induce apoptosis and inhibit angiogenesis, it remains unclear whether the antitumor activity of artemisinin is dependent on definitive molecular targets. In an attempt to investigate these molecular targets, two highly active artemisinin dimers were tested against SERCA, a possible target of artemisinin in *Plasmodium* parasites [46]. Microarray analysis of dimer treated cells identified DNA damage, iron/heme and cysteine/methionine metabolism, antioxidant response and ER stress response. However it was found that direct inhibition of SERCA was the same for active and inactive (deoxy) forms of the dimer suggesting that this plays a minimal role in ER stress induction and overall activity. This observation is not entirely surprising since artemisone fails to have any interaction with a mammalian SERCA [65]. However it was shown that the dimer, but not thapsigargin (a SERCA inhibitor) was capable of modifying reduced cysteines on SERCA [46]. In addition this evidence also suggests that direct cysteine alkylation of protein targets other than SERCA may be a prerequisite for potent activity as in *Plasmodium* parasites. 

TCTP however may be a target that both plasmodia and tumor cells have in common. Tumor cell lines with high TCTP expression were sensitive to artesunate, while a low TCTP expression was associated with resistance to artesunate [75]. TCTP represents a proliferation-related Ca^2+^-binding protein, which associates transiently with microtubules during the cell cycle. 

As in *Plasmodium*, it may be possible that heme is a mediator and a target of artemisinin in tumor cell lines. Recent evidence suggests that artemisinin has a similar mechanism to a heme interacting compound named coralyne. The absorbance spectrum of heme in combination with artemisinin shows the presence of several new intermediate complexes of artemsinin and heme. This is consistent with decomposition of the heme porphyrin ring. The cytotoxicity of both coralyne and artemisinin was found to depend on activity of synthetic heme [51]. 

Recent gene expression profiling has identified a common set of genes that were regulated by artesunate in pancreatic cancer [76]. Artesunate was identified as a novel topoisomerase inhibitor. It is thought that topoisomerase inhibitors block the ligation step of the cell cycle, generating single and double stranded breaks that harm the integrity of the genome. Introduction of these breaks subsequently lead to apoptosis and cell death. This evidence poses a question about DNA damage in *Plasmodia*. If DNA damage is evident in tumor cells then why does artemisinin not affect DNA in treatment of malaria [77]? There may be several reasons for this, firstly DNA damage in tumor cells has been shown to be dose-dependent; the dose required to kill tumor cells is higher than in *Plasmodia*. Secondly, this may suggest that artemisinin does not directly bind to DNA, but exerts its effect by an indirect oxidative stress response [78]. 

## 4. Conclusion

The molecular targets of both artemisinin in *Plasmodia* and tumor cells are still under debate. In both cases there is strong evidence to suggest that the primary activator is an iron source, be it in the form of Fe^2+^, heme or both. Mechanistic studies of fully synthetic trioxolanes and tetraoxanes that contain an endoperoxide bridge but lack other features of artemisinins have increased the complexity of the debate. It would be interesting to ascertain if these structurally simpler fully synthetic endoperoxides were as effective in tumor cells as they are in *Plasmodia*. Although protein alkylation in *Plasmodia* is well established, a single molecular target is yet to be identified which has a direct role in cell death. What is apparent is the multi-faceted nature of cellular response to artemisinin in *Plasmodia* and tumor cells. This response may explain how this drug can be used against otherwise multi-drug resistant cells in both tumors and *Plasmodia*.
